# Case report: Aplasia cutis congenita of the scalp with bone defect and an exposed sagittal sinus in a trisomy 13 newborn

**DOI:** 10.3389/fped.2023.1142950

**Published:** 2023-03-31

**Authors:** Faisal Rashed AlMatrafi, Ahmad Ayed Al-Shammari, Raed Mohamed Al Nefily, Rawan Abdulrahman AlAnazi, Abdulrahman Hamed Abdulwahab, Ahmed Sabry Ammar

**Affiliations:** ^1^ Department of Neurosurgery, College of Medicine, Imam Abdulrahman Bin Faisal University, Dammam, Saudi Arabia; ^2^ Department of Pediatrics, College of Medicine, Imam Abdulrahman Bin Faisal University, Dammam, Saudi Arabia; ^3^ Department of Pediatrics, King Fahad Hospital of the University, Al-Khobar, Saudi Arabia; ^4^ Department of Family and Community Medicine, College of Medicine, Imam Abdulrahman bin Faisal University, Dammam, Saudi Arabia; ^5^ Department of Radiology, College of Medicine, Imam Abdulrahman Bin Faisal University, Dammam, Saudi Arabia

**Keywords:** aplasia cutis congenita (ACC), trisomy 13 syndrome, bone defect, exposed sagittal sinus, patau syndrome

## Abstract

Aplasia cutis congenita (ACC) is a heterogeneous disorder with a rarely reported incidence of 0.5–1 in 10,000 births. ACC can be associated with physical defects or syndromes that may help in the diagnosis, prognosis, and further evaluation of the patient. Trisomy 13 is one of the most common fetal life-limiting diagnoses associated with ACC of membranous-type scalp. The patient was born at 35 weeks of gestation *via* a cesarean section due to fetal distress. Upon admission to our hospital, her pertinent physical examination revealed a newborn girl with dysmorphic facial features, including widely separated eyes, downward slanting of the palpebral fissure, microphthalmia, retrognathia, and low-set ears. She had an area of loss of scalp skin and skull bone with seen brain tissue and an exposed sagittal sinus that was 6 by 5 cm in size. She had a clenched fist, overlapping fingers, and rocker bottom feet. Precordium auscultation revealed medium-pitched high-grade continuous murmur heard best at the pulmonary position with a harsh machinelike quality that often radiated to the left clavicle. Laboratory investigations include basic labs, and the TORCH screen was negative. On the 9th day of life, a chromosomal analysis showed a female karyotype with three copies of chromosome number 13 (trisomy 13) in all 20 metaphase cell counts. The patient was managed with a moist gauze dressing, topical antibiotic ointment, and povidone-iodine. However, a multidisciplinary team agreed on a do-not-resuscitate (DNR) order with no further surgical intervention as the survival rate of trisomy 13 is poor. In this article, we report a case of aplasia cutis congenita of the scalp with dura and bone defect and an exposed sagittal sinus in a newborn diagnosed with trisomy 13. It emphasizes the importance of ACC-associated syndrome, which has high mortality prior to surgical intervention.

## 1. Introduction

Aplasia cutis congenita (ACC) is a focal localized congenital absence or defect in the skin with heterogeneous clinical presentations (1, 2). ACC is rare, and the reported incidence is 0.5–1 in 10,000 births (1). The pathogenesis of ACC is unknown, and it can be divided into two main pathways: the first is disruption or failure of the development of skin layers that include the epidermis, dermis, and subcutaneous fat, and the second is skin destruction in utero that was developing normally (1).

Diagnosis of ACC is clinical with appearance variability. Physical examinations may show ulcerations or erosions of the skin, which may extend to a deeper tissue, such as muscle or bone. Additionally, it may appear as an atrophic scar. Approximately 86% of aplasia cutis congenita cases involve the scalp, particulary the vertex region. Bone abnormalities in ACC are found in approximately 15–20% of cases, and the remaining majority involve the scalp (1, 2). ACC can be associated with physical defects or syndromes so it is important to identify the various clinical subtypes of ACC that will help in the diagnosis, prognosis, and further evaluation of the patient (1). ACC classification based on Frieden includes nine subtypes associated with abnormalities, inheritance patterns, and affected body areas, as shown in Table 1 (1, 2), Evers classifies ACC into four groups based on (1) chromosomal changes, (2) monogenic groups that include autosomal dominant or recessive and X-linked genetic mutations, (3) teratogenic/exogenous causes, and (4) unknown groups that include ACC with two or more congenital defects or a single congenital defect with uncertain causes (1).

**Table 1:** ACC classifications.

Type	Description
1	Scalp ACC without multiple anomalies
2	Scalp ACC with associated limb abnormalities
3	Scalp ACC with associated epidermal and organoid naevi
4	ACC overlying embryological malformations
5	ACC with associated fetus papyraceous or placental infarcts
6	ACC localized to extremities without blistering
7	ACC caused by specific teratogens
8	ACC associated with a malformation syndrome
9	Unclassified

In this article, we report cases of aplasia cutis congenita of the scalp with dura and bone defect and an exposed sagittal sinus in a newborn diagnosed with trisomy 13. It emphasizes the importance of ACC-associated syndrome, which has high mortality prior to surgical intervention.

## 2. Case description

The patient was a 23-day-old Saudi girl born to a 41-year-old lady at 35 weeks of gestation *via* a cesarean section due to fetal distress. Maternal history revealed that the mother was gravida 5 para 6 with no known chronic medical illness. She started her follow-up in a private hospital where the first ultrasound showed a single viable fetus; however, unfortunately, an anomaly scan was not performed. She took supplement medication during pregnancy after she tested positive on a pregnancy test, including folic acid, iron, and calcium, with poor adherence. There was no history of gestational diabetes or pregnancy-induced hypertension when she was screened during pregnancy follow-up. There was no history of radiation exposure. Her previous four pregnancies concluded with a normal vaginal delivery on the first occasion, the second was a cesarean section due to an abrupt placenta, the third was a cesarean section of twins, and the fourth was a cesarean section due to a previous cesarean section. However, all children were full-term and in good condition with no chronic illness. With regard to family history, the parents are not consanguineous. There is one maternal aunt with trisomy 21.

**Figure 1:**
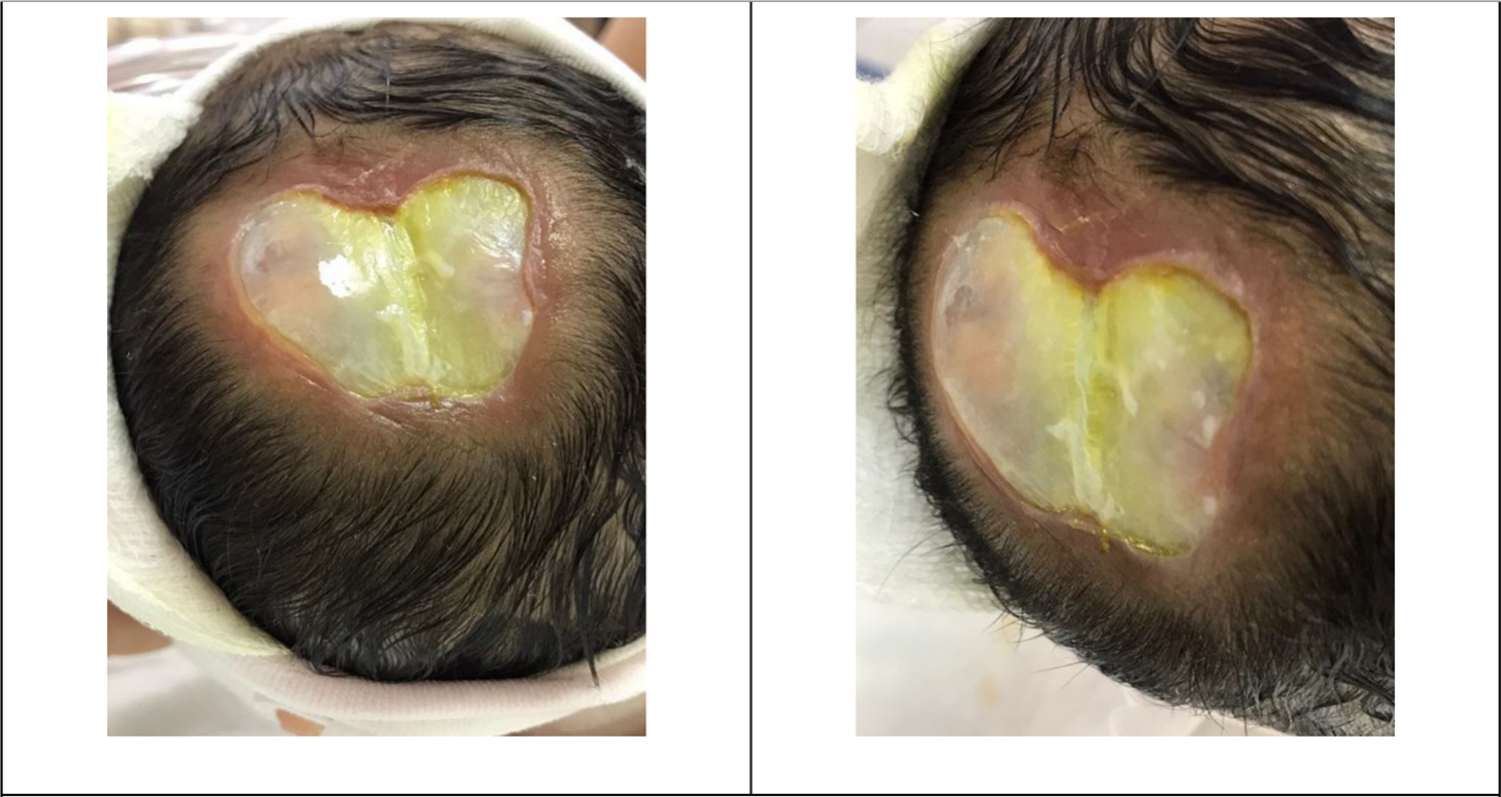
Patient’s skin defect. The newborn head showing a 6 by 5 cm full-thickness defect of the scalp, skull, and dura. An arachnoid layer covering the brain with noted brain tissue can be noticed. Also, the exposed superior sagittal sinus covered with a layer of granulation can be noticed.

The APGAR scores were 5 and 7 at 1 and 5 min at the delivery room, respectively. She was admitted to a neonatal intensive care unit (NICU) due to respiratory distress, which initially required continuous positive airway pressure (CPAP) and then intubation on the 4th day of life. She was transferred to our hospital on the 5th day of life due to limited other services and financial issues. Upon admission to our hospital, her physical examination revealed a newborn girl under a radiation warmer and intubated with connection to mechanical ventilation. Her vital signs were within acceptable values, and she had pinkish skin color and was not pale, jaundiced, or cyanosed. Her growth parameters based on age and sex were in the 10th percentile for weight, 10th percentile for length, and 10th percentile for head circumference. She had dysmorphic facial features, including widely separated eyes, downward slanting of the palpebral fissure, microphthalmia, retrognathia, and low-set ears. She had an area of loss of scalp skin and skull bone with visible brain tissue and an exposed sagittal sinus that measured 6 by 5 cm in size, as shown in Figure 1. She also had a clenched fist, overlapping fingers, and rocker bottom feet. Lung auscultation revealed bilateral vesicular breathing with equal air entry and no added sounds. Precordium auscultation revealed medium-pitched, high-grade continuous murmur heard best at the pulmonary position with a harsh machinelike quality that often radiated to the left clavicle. She had a soft and lax abdomen without tenderness or hepatosplenomegaly.

**Figure 2:**
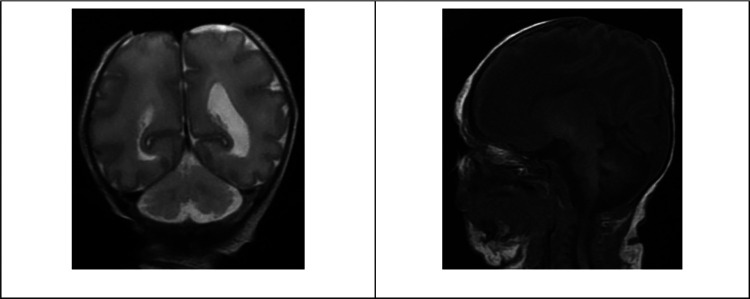
Patient head MRI. Selective images of the MRI coronal T2 and sagittal T1-weighted images showing the absence of scalp and calvarium in the parietal region with no herniation.

Laboratory investigations at the age of 5 days included basic workups within the normal range for her age. The patient’s blood group was O-positive, and the direct Coombs test (DCT) was negative. A liver function test showed total bilirubin of 13.7 mg/dl, direct bilirubin of 1.1 mg/dl, SGOT of 75 U/L, SGPT of 14 U/L, lactate dehydrogenase (LDH) of 889 U/L, GGTP of 118 U/L, total protein of 4.1 g/dl, and albumin of 2.7 g/dl. Virology screening, including the TORCH test (toxoplasma, rubella, cytomegalovirus, and herpes simplex virus), was negative. Hepatitis B surface antigen and antibody were non-reactive and the core antibody was negative. The blood culture did not show bacterial growth. On the 9th day of life, chromosomal analysis showed a female karyotype with three copies of chromosome number 13 (trisomy 13) in all 20 metaphase cell counts. Radiology investigations included head magnetic resonance imaging (MRI) and revealed absence of scalp and calvarium at the parietal region with no herniation visualized, as shown in Figure 2.

The patient was managed with a moist gauze dressing, topical antibiotic ointment, and povidone-iodine. A multidisciplinary team meeting was held that involved a neurosurgeon, a neonatologist, and pediatric genetics consultants who agreed on a do-not-resuscitate (DNR) order with no further surgical intervention as the survival rate of trisomy 13 is poor along with associated skull bone defect.

## 3. Discussion

In 1767, Gordon was the first to report aplasia cutis congenita (1). Chromosomal analysis is recommended for any newborn with scalp ACC with high consideration if there are other congenital anomalies as genetic causes of ACC, as shown in Table 2 (3). Such an associated anomaly is trisomy 13, which is one of the most common fetal life-limiting diagnoses, with a prevalence of 1.68 per 10,000 births (4). The median survival period for live births with trisomy 13 is approximately 10 days (5). Anomalies associated with trisomy 13 include 34% with major cardiac anomalies, 25% with orofacial clefts, and 11% with nervous system anomalies (5). ACC of scalp type is a common association with trisomy 13, but the bone association is rare (1).

**Table 2:** ACC-associated congenital anomalies.

Chromosomal	Patau syndrome, Pallister–Killian syndrome, Wolf–Hirschhorn syndrome
Autosomal dominant	Adams–Oliver syndrome, autosomal dominant ACC, ectrodactyly-ectodermal dysplasia-clefting syndrome, ectodermal dysplasia, scalp–ear–nipple syndrome
Autosomal	Recessive autosomal recessive ACC, Johanson–Blizzard syndrome, setleis syndrome, ectodermal dysplasia-clefting syndrome, epidermolysis bullosa
X-linked	Goltz–Gorlin syndrome (focal dermal hypoplasia), MIDAS (microphthalmia, dermal aplasia, and sclerocornea) syndrome
Teratological/exogenous	Alcohol, cocaine, marijuana, methimazole, misoprostol, congenital infections (herpes simplex, rubella, varicella), amniotic band disruption complex

Skull or dura defect in ACC results in brain and sagittal sinus exposure. Such exposure increases the risk of hemorrhage, infection, and sagittal sinus thrombosis. The reported fatality of ACC is 20–50% (6). The preoperative evaluation includes history, physical examination, and radiological investigation by three-dimensional facial bone computed tomography (CT) and magnetic resonance imaging (MRI) of the brain (6). The guiding prioritization criterion and the choice of intervention remain controversial and vague. However, it is important for neurosurgeons to decide promptly whether to perform early surgical intervention or to proceed with conservative care (6). For example, small lesions with noninjured dura and small bone defects that do not overlie the superior sagittal sinus can be managed conservatively by a gauze dressing with saline drips, topical antibiotic ointment, povidone-iodine, and silver sulfadiazine, which hopefully will heal gradually with re-epithelization. The goal of such management is to ensure and maintain a moist healing environment to avoid eschar formation and minimize bleeding risk. Meanwhile, large defects with full thickness involvement of scalp and bone require more time for complete closure as they require a longer procedure (7). However, large scalp and skull defects can be managed conservatively with complete healing, as has been reported (6). Surgical intervention indication includes enlarged vein exposure, associated dura defect, and brain exposure. Such surgical options include split-thickness or full-thickness skin grafts, scalp rotation flaps, pericranial flaps, split rib grafts with latissimus dorsi muscle flaps, and tissue expansion (7).

Surgical management complications include hemorrhage, graft or flap loss, infection, donor site morbidity, and anesthesia-related complications (1, 6). However, conservative management may develop complications, such as massive hemorrhage, cerebrospinal fluid leak, central nervous system infection, and sepsis. In contrast, in cases of dural defects, the patient may have herniation of the brain with parenchymal injury and cerebral necrosis (6, 7). Additionally, if ACC occurs due to a genetic disorder, it will further burden the surgical complication if there are other comorbidities.

In conclusion, the history and physical examination of ACC can guide healthcare providers to the underlying pathophysiology and further evaluations and treatments can be performed if needed. Family counseling is important to provide understandable family values and goals, as well as to achieve the expected outcomes and disease trajectories (4). The case emphasizes the importance of ACC management, a comprehensive approach that involves the clinical status of the patient to determine the best surgical and non-surgical management.

## Data Availability

The original contributions presented in the study are included in the article/Supplementary Material. Further inquiries can be directed to the corresponding author.
